# Upregulated CD8^+^ MAIT cell differentiation and *KLRD1* gene expression after inactivated SARS-CoV-2 vaccination identified by single-cell sequencing

**DOI:** 10.3389/fimmu.2023.1174406

**Published:** 2023-08-15

**Authors:** Xiaowen Dou, Mian Peng, Ruiwei Jiang, Weiqin Li, Xiuming Zhang

**Affiliations:** ^1^ Medical Laboratory of the Third Affiliated Hospital of Shenzhen University, Shenzhen, China; ^2^ Department of Critical Care Medicine, The Third Affiliated Hospital of Shenzhen University, Shenzhen, China; ^3^ Department of Critical Care Medicine, Jinling Hospital, Affiliated Hospital of Medical School, Nanjing University, Nanjing, China

**Keywords:** CD8^+^ MAIT cell differentiation, *KLRD1* gene, single-cell sequencing, inactivated SARS-CoV-2 vaccine, COVID-19

## Abstract

**Background:**

The primary strategy for reducing the incidence of COVID-19 is SARS-CoV-2 vaccination. Few studies have explored T cell subset differentiation and gene expressions induced by SARS-CoV-2 vaccines. Our study aimed to analyze T cell dynamics and transcriptome gene expression after inoculation with an inactivated SARS-CoV-2 vaccine by using single-cell sequencing.

**Methods:**

Single-cell sequencing was performed after peripheral blood mononuclear cells were extracted from three participants at four time points during the inactivated SARS-CoV-2 vaccination process. After library preparation, raw read data analysis, quality control, dimension reduction and clustering, single-cell T cell receptor (TCR) sequencing, TCR V(D)J sequencing, cell differentiation trajectory inference, differentially expressed genes, and pathway enrichment were analyzed to explore the characteristics and mechanisms of postvaccination immunodynamics.

**Results:**

Inactivated SARS-CoV-2 vaccination promoted T cell proliferation, TCR clone amplification, and TCR diversity. The proliferation and differentiation of CD8^+^ mucosal-associated invariant T (MAIT) cells were significantly upregulated, as were *KLRD1* gene expression and the two pathways of nuclear-transcribed mRNA catabolic process, nonsense-mediated decay, and translational initiation.

**Conclusion:**

Upregulation of CD8^+^ MAIT cell differentiation and *KLRD1* expression after inactivated SARS-CoV-2 vaccination was demonstrated by single-cell sequencing. We conclude that the inactivated SARS-CoV-2 vaccine elicits adaptive T cell immunity to enhance early immunity and rapid response to the targeted virus.

## Background

Coronavirus disease 2019 (COVID-19) has attracted global attention since its emergence in December 2019 in Wuhan, China (1–3) due to its high transmissibility and rapidly surging case numbers (4, 5). On 30 January 2020, by which time severe acute respiratory syndrome coronavirus 2 (SARS-CoV-2) had infected nearly 10,000 people and had caused over 200 deaths, the World Health Organization (WHO) declared the COVID-19 outbreak a public health emergency of international concern (6, 7). Soon afterward, on 11 March 2020, the WHO upgraded its classification of the COVID-19 outbreak to a global pandemic (8).

The primary strategy for reducing the incidence of COVID-19 is SARS-CoV-2 vaccination. Ongoing research on SARS-CoV-2 vaccine mechanisms of action is comprised primarily of qualitative and quantitative studies of antibody and cytokine responses (9–13). Only a few studies have explored T cell subset differentiation and gene expression. Single-cell sequencing is a sensitive method for in-depth analysis of the cellular and genetic mechanisms of vaccine response. Single-cell sequencing has identified an antigen-specific cellular basis of BNT 162b2 mRNA vaccine-induced immunity (14) and cell type-specific interferon responses to an Ad5-nCoV adenovirus vaccine that enhanced cellular immunity (15). However, single-cell sequencing has rarely been used to explore the mechanism of inactivated SARS-CoV-2 vaccines. In this study, single-cell sequencing was used to analyze the dynamics of T cell-mediated immunity and transcriptome gene expression for 3 months in three healthy Chinese adults who received three doses of inactivated SARS-CoV-2 vaccine.

## Methods

### Participants

Healthy adult volunteers were recruited on 8-14 January 2021. Inclusion criteria were an age of 18–59 years and good health without underlying diseases. Exclusion criteria were age younger than 18 years or older than 60 years, underlying diseases, serious adverse reactions during vaccination, illnesses requiring hospitalization, pregnancy, a history of miscarriage, or withdrawal from the study for any reason during follow-up. This study was approved by the Research Ethics Committee of the Third Affiliated Hospital of Shenzhen University and written informed consent was obtained before enrollment (The EC approval number: 2021-LHQRMYY-KYLL-033).

### Sample collection and preparation

A total of 1 mL blood samples were collected from each participant at four time points: immediately before the first SARS-CoV-2 vaccination dose, 14 and 90 days after the second dose, and 90 days after the third dose (V0, V1, V2, and V3, respectively). Peripheral blood mononuclear cells (PBMCs) were extracted by density gradient centrifugation. The whole blood sample was diluted with phosphate-buffered saline (PBS, Solarbio, Beijing, China) at a ratio of 1:1. The diluted sample was added to a tube with 2/3 volume of Ficoll-Pague PLUS lymphocyte separation medium (GE Healthcare, Sweden). Cells were divided into three layers because of different sizes and densities after centrifugation at 400g for 35 min. The supernatant was removed, and the intermediate cell suspension layer was transferred into a 15 ml centrifuge tube, supplemented with PBS, and centrifuged at 300g for 7 min. The supernatant was again discarded, and the pellet was washed twice and resuspended in PBS to obtain PBMCs. PBMCs were extracted and frozen at -80°C. PMBC concentrations and activities were measured before the study began. PBMCs were taken from the -80°C freezer and thawed. The cell mixture sample was stained with 0.4% Trypan blue solution (Sigma, UK), and viable cells were counted under a microscope (ECLIPSE Ts2, Nikon, Japan). When the final concentration was 2 × 10^5^ cells/mL and cell viability exceeded 85%, subsequent processing was performed.

### Library preparation and single-cell T cell receptor sequencing

Cell suspensions (2×10^5^ cells/ml, 100µl) were loaded into microfluidic devices (Matrix1.0.1) and the separation of single cells was completed according to the principle of Poisson distribution. scTCR-seq libraries were constructed following the protocol of GEXSCOPE Single Cell Immuno-TCR Kit (Biotechnologies). Specifically, poly(A) tails and TCR regions of mRNA were captured by magnetic beads with molecular markers. Cells and mRNA were labeled after the cells were lysed. The magnetic beads in the chip were collected, and mRNAs were reverse-transcribed into complementary DNA (cDNA) and amplified. After local cDNAs were fragmented and spliced, transcriptome sequencing libraries suitable for the Illumina sequencing platform were constructed. The remaining cDNA was enriched to the immune receptor (TCR), and TCR sequencing libraries suitable for the Illumina sequencing platform were constructed by PCR amplification of the enriched products. Finally, sequencing of the libraries was performed on Illumina Nova 6000, with a pair-end length of 150 bp.

### TCR V(D)J sequencing and analysis

The Cell Ranger (v4.0.0) vdj (variable, diversity, joining region) pipeline was used to analyze TCR clonotype, with Genome Reference Consortium Human Genome Build 38(GRCh38) as reference. After the analysis, a TCR diversity metric of clonotype frequency and barcode information was acquired. For TCR, only cells with one productive TCR α-chain (TRA) and one productive TCR β-chain (TRB) were retained for subsequent analysis. Each unique TRA(s)-TRB(s) pair was defined as a clonotype. If one clonotype was present in at least two cells, cells harboring this clonotype were considered as clonal and the number of cells with such pairs indicated the degree of clonality of the clonotype (16). Clonotype diversity was calculated with Chao1.

### Primary analysis of raw read data

Using an internal pipeline (a data conversion process), raw reads from scRNA-seq were converted into gene expression matrices. Cell barcodes and unique molecular identifiers (UMI) were extracted by first removing low-quality data of raw reads with FastQC v0.11.4 and fastp (17), and then trimming poly-A tails and adapter sequences with Cutadapt (18). UMI and gene counts of each cell were then acquired with featureCounts v1.6.2 (19) after the reads were mapped to the reference genome GRCh38 by using STAR v2.5.3a (20). Expression matrix files were thus generated.

### Quality control, dimension-reduction, and clustering

During the quality control process, the gene expression matrix was filtered after excluding the following cells: cells with gene count top 2% or < 200, cells with top 2% UMI count, cells with 50% mitochondrial content, and cells in which genes were expressed in < 5 cells. After quality control, dimension reduction and clustering were performed by using Seurat v3.1.2 (21). All gene expressions were then normalized and scaled with NormalizeData and ScaleData functions, and the top 2,000 variable genes were selected for principal component analysis with FindVariableFeatures. Cells were separated into multiple clusters according to the top 20 principle components by using FindClusters. Harmony (22) was used to remove the batch effect between samples. Finally, two-dimensional visualization of cells was achieved by using uniform manifold approximation and projection (UMAP).

### Inference of cell differentiation trajectories

Cell differentiation trajectories were reconstructed by Monocle2 v2.22.0. Cell spatiotemporal differentiation sequencing was performed by evaluating highly variable genes. FindVariableFeatures and dimensional reduction were performed by DDRTree. Finally, the trajectories were visualized by using the plot_cell_trajectory function.

### Differentially expressed gene analysis

The genes expressed in > 10% of the cells in a cluster and with an average log(fold change) value > 0.25 were selected and identified as DEGs by using the Seurat FindMarkers function based on the Wilcox likelihood-ratio test with default parameters. The cell type annotation of each cluster was displayed with dot plots/violin plots by using Seurat DotPlot/Vlnplot, according to the expression of canonical markers found in the DEGs and knowledge from the literature. Cells expressing markers of different cell types were identified as doublets and removed during subsequent quality control.

### Pathway enrichment analysis

Pathway enrichment analysis was performed by using Gene Ontology (GO) analysis together with the clusterProfiler R package (23), aiming to investigate the functions of DEGs. Molecular function, biological process, and cellular component categories in GO gene sets were used as references. Protein-protein interactions of DEGs in each cluster were predicted according to the interactions between the known genes and the relevant GO terms in StringDB v1.22.0. Pathways with *p*_adj < 0.05 were considered significantly enriched.

### Statistical analysis

Statistical analyses and visualization were performed with the R package (R Foundation for Statistical Computing, Vienna, Austria). Data were presented as mean ± standard deviation. Comparisons between the two groups were analyzed using a two-sample Student’s *t*-test. *P <*0.05 was considered statistically significant.

## Results

### Participants

Three subjects were recruited, including one man and two women, with an average age of 33.00 ± 13.08 years and an average body mass index of 20.13 ± 2.40 kg/m^2^. All subjects received three doses of inactivated SARS-CoV-2 vaccine, with a 4-week interval between the first and second dose and a 32-week interval between the second and third dose. None of the subjects experienced serious adverse reactions.

### PBMC concentration and activity

As shown in **Table 1**, the final PBMC concentrations were > 2×10^5^ cells/mL at all time points, and cell viability exceeded 85%. All PBMCs were qualified; thus subsequent procedures were processed.

**Table 1:** PMBC concentrations and activities of each subject at each time point.

Each study time point of each subject	Cell concentration (cells/mL)	Cell activity (%)
S1_V0	1.55×10^6^	90.41
S1_V1	1.75×10^6^	94.60
S1_V2	5.51×10^5^	95.50
S1_V3	1.03×10^6^	90.40
S2_V0	1.11×10^6^	90.56
S2_V1	9.48×10^5^	85.87
S2_V2	1.20×10^6^	93.91
S2_V3	1.47×10^6^	96.40
S3_V0	1.62×10^6^	91.11
S3_V1	5.87×10^5^	94.79
S3_V2	1.63×10^6^	93.94
S3_V3	1.14×10^6^	96.10

S1, S2, and S3 refer to the three study subjects.

### Cell type identification

**Figure 1:**
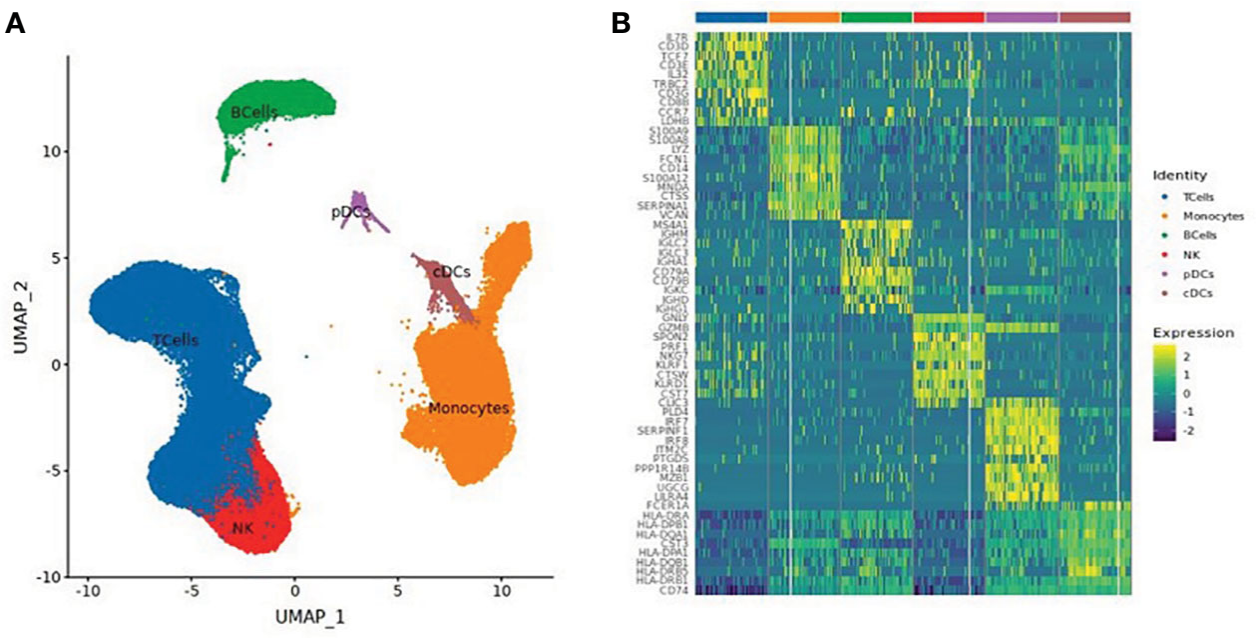
**(A)** UMAP Clustered by cell type coloring for all cells. **(B)** Heatmap of the top 10 differential genes of each cell type.

A total of 130,082 PBMCs were obtained from the 12 blood samples taken from the three subjects at the four time points. After clustering, the cells were classified into six cell types, including B cells (corresponding B cell genes were *MS4A1*, *CD79A, CD79B*, *JCHAIN*, *MZB1*, *IGHG1*, and *IGHA1*), T cells (*CD2*, *CD3D*, *TRAC*, *TRBC2*, *KLRD1*, and *NKG7*), NK cells (*KLRD1*, *KLRF1*, *NKG7*, *NCR1*, *XCL1*, and *CD3D*), monocytes (*CD14*, *FCN1*, *VCAN*, *FCGR3A*, and *IFITM3*), conventional dendritic cells (cDCs) (*CD1C*, *FCER1A*, *XCR1*, and *CLEC10A*), and plasmacytoid dendritic cells (pDCs) (*IL3RA*, *CLEC4C*, and *LILRB4*), as shown in **Table 2** and **Figure 1**. The numbers of NK and especially T cells increased significantly after vaccination. However, no significant expansions of B cells, monocytes, cDCs, or pDCs were observed. Furthermore, the numbers of these cells at the V3 time point had decreased from V0 baseline values.

**Table 2:** Quantities of six cell types obtained by clustering from 130,082 cells of three individuals at different time points after SARS-CoV-2 vaccine inoculation.

	B cells	T cells	NK cells	Monocytes	cDCs	pDCs
S1_V0	791	4837	296	2061	47	44
S2_V0	1196	1183	1272	5298	443	97
S3_V0	642	1034	606	2975	143	69
S1_V1	575	5483	1498	2775	115	76
S2_V1	650	1254	1316	5382	596	231
S3_V1	864	2875	1578	4962	203	230
S1_V2	1111	11036	2033	2167	156	99
S2_V2	725	5711	925	1787	195	65
S3_V2	1396	11292	1846	2069	136	73
S1_V3	576	4976	915	2522	161	71
S2_V3	785	6472	2045	3182	199	61
S3_V3	715	6838	723	3093	173	56

### Subdivision of T cell subsets

**Figure 2:**
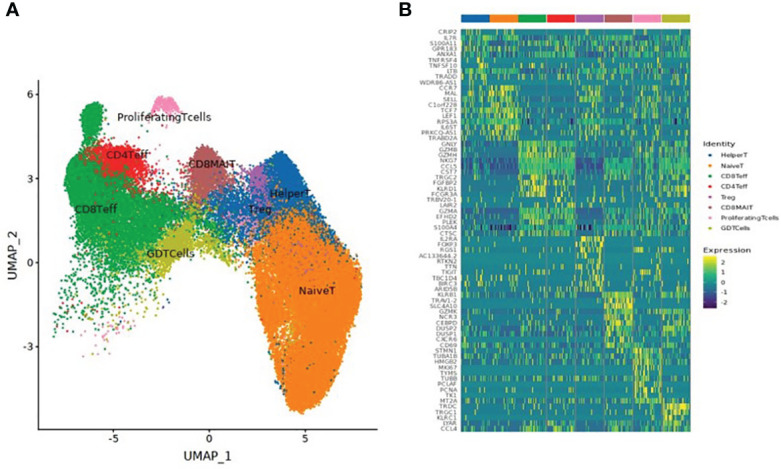
**(A)** UMAP clustering of T cell subsets. **(B)** Heatmap of the top 10 differential genes of each T cell subset.

A total of 61,599 T cells were classified into 8 T cell subsets: naïve T cells, CD4^+^ effector T cells, CD8^+^ effector T cells, CD8^+^ mucosal-associated invariant T (CD8^+^ MAIT) cells, helper T cells, regulatory T cells (Treg), Gamma Delta T cells (GDT cells), and proliferating T cells, as shown in **Table 3** and **Figure 2**.

**Table 3:** Quantities of eight T cell subsets obtained by clustering from 61,599 T cells of three individuals at different time points after SARS-CoV-2 vaccine inoculation.

T cell subsets	V0	V1	V2	V3
Naïve T cells	3655	3665	14236	8548
CD4^+^ effector T cells	218	500	1010	837
CD8^+^ effector T cells	1440	3232	5935	3769
D8^+^ MAIT cell	331	443	1429	911
Helper T cell	820	846	3002	2246
Treg	179	151	563	461
GDT cells	286	445	1074	657
Proliferating T cells	64	138	250	258

### Dimension reduction analysis of TCR-amplified clonotypes

**Figure 3:**
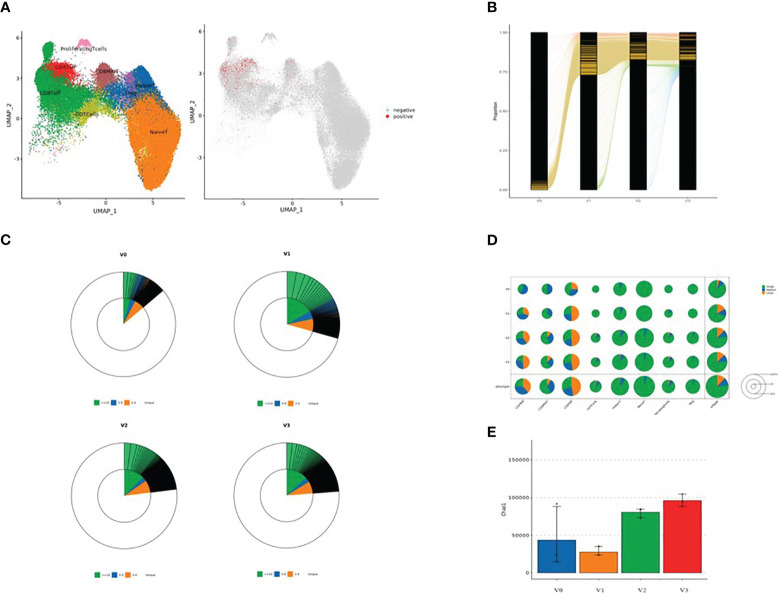
**(A)** Dimensional reduction analysis of TCR-amplified clonotypes (red dots indicate T cells with clonotype expansion and gray dots indicate T cells without clonotype expansion); **(B)** Overall dynamic proportion of TCR clonotype amplification at different time points; **(C)** Proportion of TCR clonotype frequencies (large, medium and single) at time points V0, V1, V2, and V3 (green: clonotype frequency ≥10; blue: clonotype frequency 5-9; yellow: clonotype frequency 2-4; and white: clonotype frequency single); **(D)** TCR clonotype amplification of different T cell subsets at four time points: V0, V1, V2, and V3 (green: single, blue: medium, and yellow: large). The area of each circle represents the number of cells; the larger the circle, the greater the number of T cell subsets with TCR-amplified clonotypes; and **(E)** TCR diversity analysis (V0: blue, V1: yellow, V2: green, and V3: red).

After dimension reduction analysis, TCR clonotypes were classified into large, medium, and single groups according to the frequency of amplified clonotypes. Large, medium, and single clonotype frequencies were defined as >10, >1 and ≤10, and 1, respectively. The results are shown in **Figure 3**. In **Figure 3A**, red and gray dots indicate T cells with or without clonotype expansion, respectively. **Figure 3B** shows the overall dynamic proportion of TCR clonotype amplification at different time points. **Figure 3C** shows the proportion of TCR clonotype large, medium, and single frequencies at different time points. **Figure 3D** shows the TCR clonotype amplification of different T cell subsets at different time points. **Figure 3E** illustrates the TCR diversity analysis results. T cell subsets with significant TCR clonotype amplification were CD4^+^ effector T cells, CD8^+^ effector T cells, and CD8^+^ MAIT cells. The proportion of large clone amplification in these three T cell subsets was higher after inoculation. As shown in **Figure 3E**, TCR diversity was higher at V2 and V3 than before inoculation (although *P >*0.05), with the highest value at V3, suggesting that TCR diversity increased after inactivated SARS-CoV-2 vaccination.

### CD8^+^ MAIT cell differentiation trajectories

**Figure 4:**
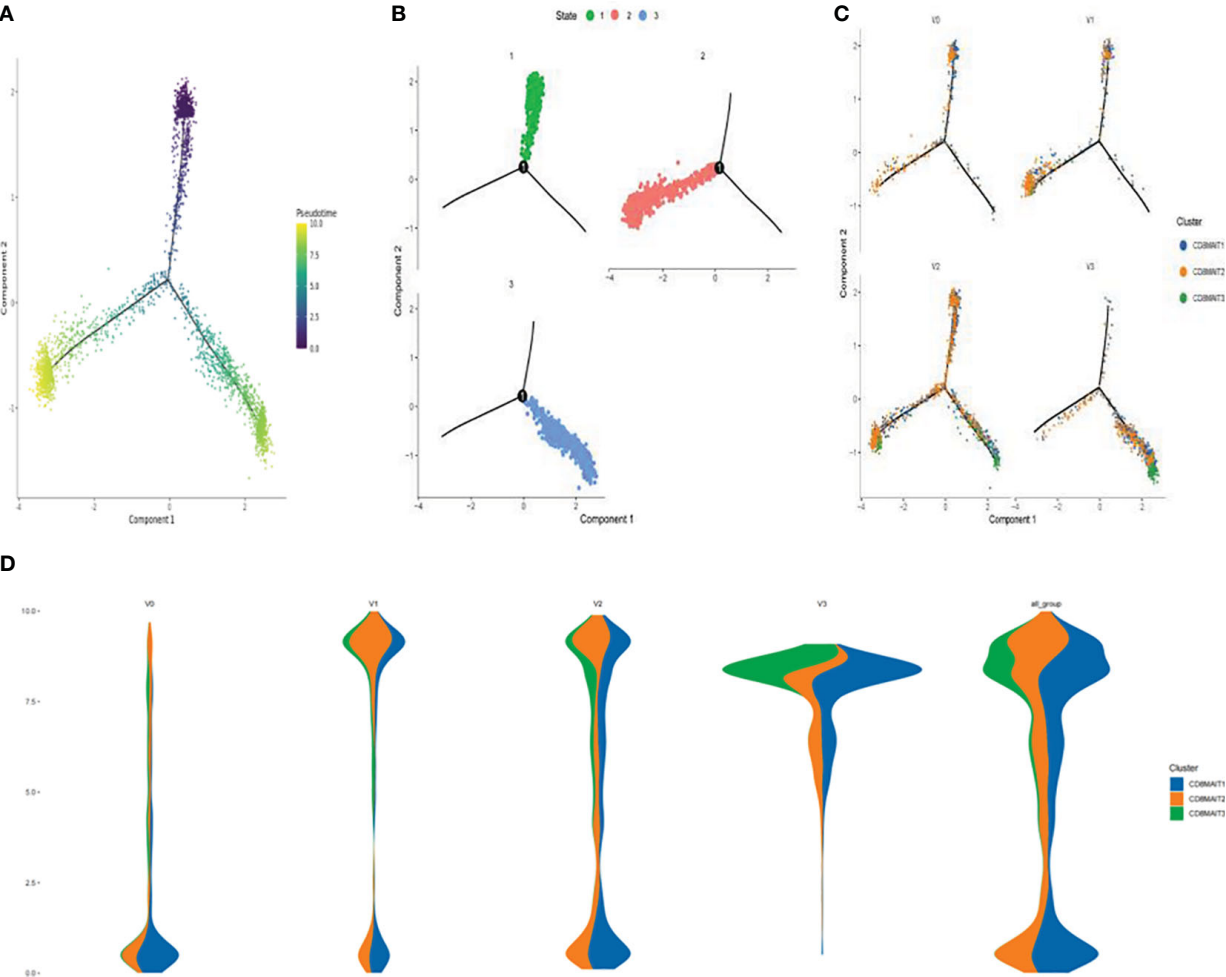
Differentiation trajectory of CD8^+^ MAIT cells **(A)** Pseudotime trajectory of CD8^+^ MAIT cells; **(B)** Three stages of CD8^+^ MAIT cells differentiation; **(C)** Trajectory analysis of CD8^+^ MAIT cells from different time points V0, V1, V2, and V3; and **(D)** Differentiation process of CD8^+^ MAIT cells at different inoculation time points.

The results of the pseudo-time sequence analysis of CD8^+^ MAIT cells are shown in **Figure 4**. **Figure 4A** shows the overall trajectory of CD8^+^ MAIT cell differentiation. **Figure 4B** shows the three stages of CD8^+^ MAIT cell differentiation. **Figure 4C** shows the trajectory analysis of CD8^+^ MAIT cells from different time points V0, V1, V2, and V3. **Figure 4D** (spindle diagram) shows the CD8^+^ MAIT cell differentiation process at each time point. CD8^+^ MAIT cells were typically within a relatively late stage of differentiation at V0. However, these cells differentiated significantly after vaccination. A large proportion of CD8^+^ MAIT cells with relatively advanced differentiation appeared at V1. At V2, CD8^+^ MAIT cells continued to differentiate. At the final V3 time point, after the third dose, many CD8^+^ MAIT cells had reached the differentiation endpoint.

### DEG analysis

**Figure 5:**
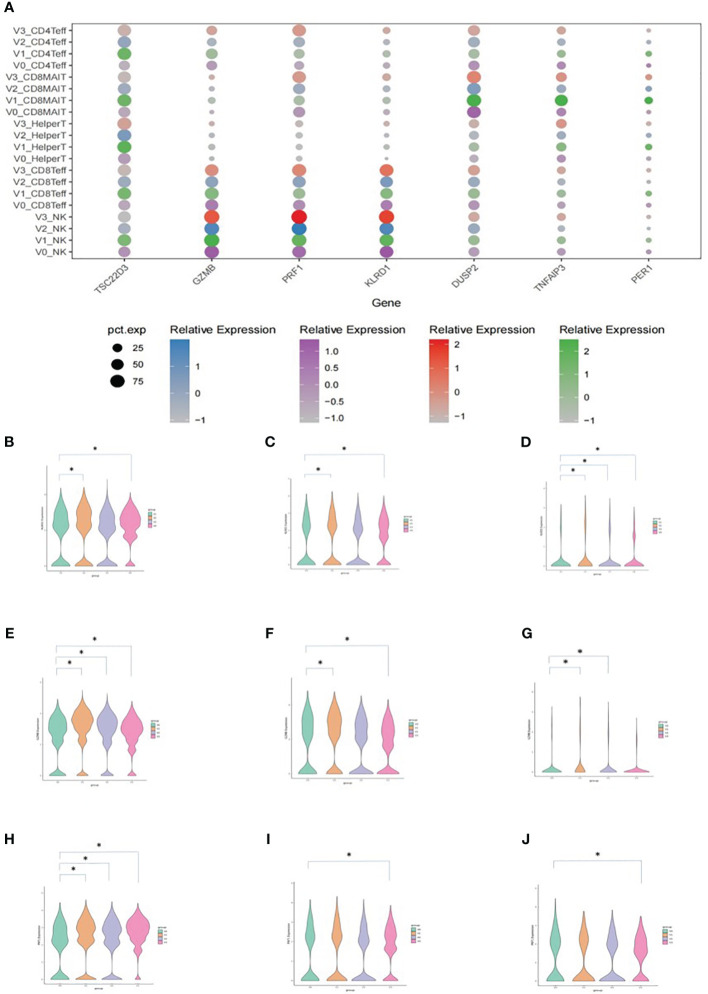
Average expression levels of DEGs. **(A)** Dotplot expressing the average expression of *TSC22D3*, *GZMB*, *PRF1*, *KLRD1*, *DUSP2*, *TNFAIP3*, and *PER1* in T cell subsets. The X-axis indicates the DEGs and the Y-axis shows different T cell subsets at each time point. V0: purple, V1: green, V2: blue, and V3: red. The size of the dot indicates the proportion of cells that expressed the genes in its cell subset, and the shade of the color of the dot indicates the average level of gene expression in all cells. **(B)** Vlnplot of the average *KLRD1* expression in NK cells. **(C)** Vlnplot of the average *KLRD1* expression in CD8^+^ effector cells. **(D)** Vlnplot of the average *KLRD1* expression in CD8^+^ MAIT cells. **(E)** Vlnplot of the average *GZMB* expression in NK cells. **(F)** Vlnplot of the average *GZMB* expression in CD8^+^ effector cells. **(G)** Vlnplot of the average *GZMB* expression in CD8^+^ MAIT cells. **(H)** Vlnplot of the average *PRF1* expression in NK cells. **(I)** Vlnplot of the average *PRF1* expression in CD8^+^ effector cells. **(J)** Vlnplot of the average *PRF1* expression in CD8^+^ MAIT cells. In panels **(B-J)**, the X-axis shows the four time points and the Y-axis shows the average expression levels of the corresponding gene. V0: green, V1: yellow, V2: purple, and V3: red. *P<0.05.

The results of the DEG analysis are shown in **Figure 5**. DEGs included *TSC22D3*, *GZMB*, *PRF1*, *KLRD1*, *DUSP2*, *TNFAIP3*, and *PER1* (**Figure 5A**). Average expressions of *KLRD1* in NK cells, CD8^+^ effector T cells, and CD8^+^ MAIT cells were significantly increased after vaccination when compared with V0 baselines. The average expressions of *GZMB* and *PRF1* in NK cells, CD8^+^ effector T cells, and CD8^+^ MAIT cells were also significantly increased after vaccination (*P<0.05).

### Enrichment pathway analysis

**Figure 6:**
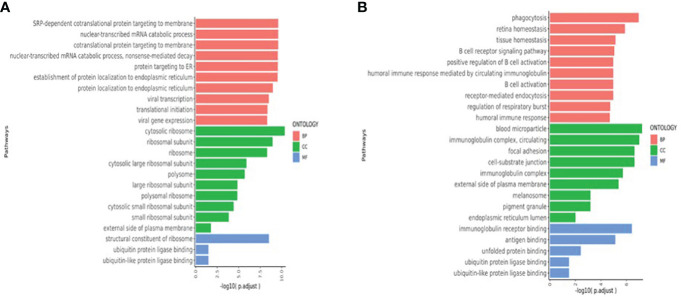
(**A**)T cell enriched upregulated pathways (V1V2V3 *vs.* V0). (**B**) B-cell enriched upregulated pathways (V1V2V3 *vs.* V0). The Y-axis shows pathways that were significantly enriched at V1, V2, and V3 time points compared with V0 baselines and the X-axis shows the *P*_adjust value compared between pathways. The larger the *P*_adjust value, the more significant the statistical difference. BP, biological processes; CC, cellular component; MF:,molecular function.

Pathways that were significantly enriched at V1, V2, and V3 were selected **(** **Figure 6** **)**. The Y-axis shows pathways that were significantly enriched at the postvaccination time points when compared with V0 baselines, and the X-axis shows the *P*_adjust value compared between pathways. The larger the *P*_adjust value, the more significant the statistical difference. In the T cell biological process pathways (**Figure 6A**), we found that meaningful enrichment pathways were significantly upregulated. These included the nuclear-transcribed mRNA catabolic process, nonsense-mediated decay, and translational initiation pathways, which play important roles in limiting viral replication and regulating post-vaccination immunity. Furthermore, in the B-cell biological process pathways (**Figure 6B**), several B-cell and B-cell receptor enrichment pathways, which included B cell receptor signaling pathway, positive regulation of B cell activation, B cell activation, and humoral immune response pathways, were all significantly enhanced.

## Discussion

The deployment of SARS-CoV-2 vaccines marked an important milestone in the COVID-19 pandemic (24–26). To date, the WHO reported that more than 100 vaccine candidates have been in development globally and 26 have been evaluated in phase III clinical trials. Although SARS-CoV-2 vaccines prevent COVID-19 (27–33), vaccine-breakthrough cases still occur in fully vaccinated individuals (34–38). Several variants of concern have emerged, which include Alpha (501Y.V1 with GISAID nomenclature or B.1.1.7 with PANGO nomenclature), Beta (501Y.V2 or B.1.351), Gamma (501Y.V3 or P1),and Delta (G/478K.V1 or B.1.617.2). Variants of concern are generally associated with higher transmission, mortality, and breakthrough infections than the original strain or D614G variant.

Different SARS-CoV-2 vaccines have demonstrated varying protective efficacies. A meta-analysis (39) showed that after full vaccination, mRNA vaccine efficacy against symptomatic SARS-CoV-2 infection was 89-100% against unsequenced strains, 88-100% against Alpha, 76-100% against Beta/Gamma, and only 47.3-88% against Delta; while the adenovirus-vectored vaccine AZD1222 was 74.5% protective against Alpha and 67% against Delta. These results suggest that mRNA vaccines and AZD1222 are effective in preventing symptomatic SARS-CoV-2 infection against the original strain and Alpha and Beta variants, but less effective against the Delta strain. Meanwhile, vaccine efficacy decays after completion of the immunization series. Antibody levels after BNT162b2, mRNA-1273, and Ad26.COV2.S vaccinations were sustained for at least 6 months but then decreased over time. At 6 months, neutralizing antibody activities against Alpha, Gamma, Delta, and Epsilon were maintained, but declined against Beta in half of the participants of mRNA-1273 vaccination. Observa- tional studies stratified by time since vaccination showed that the efficacy of mRNA vaccine and AZD1222 vaccine in preventing Delta infection decreased significantly at 4-6 months after inoculation (42-57% and 47.3%, respectively). In the USA, mRNA vaccine efficacy against symptomatic SARS-CoV-2 infection decreased from 94.3% in June to 65.5% in July 2021.

Because the efficacies and durations of protection of different COVID-19 vaccines against particular SARS-CoV-2 strains have varied, vaccine targets, mechanisms, and duration of protection must be explored further. In this study, we used single-cell sequencing to track the immune status after three doses of inactivated SARS-CoV-2 vaccine. Results revealed the T cell dynamics, characteristics, and mechanisms of human immune dynamics.

Our previous research work (40) showed that serum IgM, IgG, and neutralizing antibody titers peaked on the 14th day after the second dose of inactivated SARS-CoV-2 vaccine and decreased gradually thereafter. Therefore, in the present study, we chose four time points: V0 (pre-vaccination), V1(14 days after 2nd inoculation), V2 (90 days after the 2nd inoculation), and V3 (90 days after the 3rd inoculation) to explore the changes of immune statuses of healthy adults during the course of three doses of SARS-CoV-2 inactivated vaccine.

Single-cell RNA sequencing is a powerful tool for elucidating transcriptome gene expression and dynamics of cell subsets (41). Changes in the immune landscape at different time points after inactivated SARS-CoV-2 vaccination were analyzed by first determining the different proportions of cell subsets, then tracking cell differentiation trajectories through pseudo-time sequence analyses, and, finally, by analyzing DEGs and pathway enrichment between cells of amplified and unamplified clonotypes, thereby exploring the dynamics of transcriptome gene expression.

The serum-neutralizing antibody titer is usually taken as an important marker of the immunogenicity of an anti-viral vaccine. Zhang et al. (42) found that transcription levels of PBMC were changed 14 days after the first dose of inactivated SARS-CoV-2 vaccine in 13 healthy participants while serum-neutralizing antibody concentrations remained very low. On day 28, upon the second vaccine dose, the subjects’ PBMC transcriptomics achieved an immune status similar to natural immunity, suggesting that PBMC single-cell sequencing is more sensitive than classical neutralizing antibody assays. Horns et al. (43) studied influenza vaccine response and found that single-cell transcriptional profiling reveals a program of memory B cell activation characterized by *CD11c* and *T-bet* expression associated with clonal expansion and differentiation toward effector function. Kong et al. (44) used single-cell transcriptomic measurements to demonstrate that Bacillus Calmette-Guérin vaccination (BCG) reduces systemic inflammation and to identify a group of genes that are putatively responsible for the non-specific protection conferred by the Bacillus Calmette-Guérin (BCG) vaccination. Because single-cell sequencing is not only more sensitive in evaluating immunogenicity and can further explore the molecular and cellular characteristics and mechanisms of the immune response, we chose single-cell sequencing as our research method.

Our study showed that the numbers of NK and especially T cells increased significantly after vaccination. However, the numbers of B cells, monocytes, cDCs, and pDCs were not increased. Moreover, the numbers of these cells at the V3 time point were reduced when compared with pre-inoculation baselines.

Our study showed that the number of T cell subsets increased significantly on the 14th day after the 2nd dose, 90 days after the 2nd dose, and 90 days after the 3rd dose of inactivated SARS-CoV-2 vaccination. Subsets included naïve T cells, CD4^+^ effector T cells, CD8^+^ effector T cells, CD8^+^MAIT cells, helper T cells, Tregs, GDT cells, and proliferating T cells **(** **Table 3** and **Figure 2** **)**. Similar to our study, Mateus et al. (45) evaluated subjects in multiple age groups who received low-dose (25ug) mRNA-1273 COVID-19 vaccine and found that vaccine-generated spike-specific CD4^+^ and CD8^+^ T cell immune memory 6 months after the second dose of the vaccine was comparable in quantity and quality to natural immunity.

We further performed dimension reduction analysis of TCR-amplified clonotypes and found significant amplification of TCR clonotypes after inactivated SARS-CoV-2 vaccination, especially in CD4^+^ effector T cells, CD8^+^ effector T cells, and CD8^+^ MAIT cells. We observed a trend toward increased TCR diversity at 90 days after the second inoculation and 90 days after the third inoculation when compared to the pre-vaccination baseline (**Figure 3**). TCR is the characteristic surface marker of T cells, whose function is antigen recognition. TCR is a heterodimer composed of α and β peptide chains. Each peptide chain contains variable (V) and constant (C) regions. The antigen specificity of TCR is conferred by the V region, in which there are three highly variable regions, namely, complementarity determining regions (CDR) 1, CDR2, and CDR3. When TCR recognizes a peptide-MHC complex, CDR1 and CDR2 bind to the lateral wall of the MHC molecular antigen-binding slot, while CDR3 binds directly to the antigen-binding peptide, determining the antigen-binding specificity of TCR. Crucial to immune function is the ability to recognize the millions of antigens that may be presented *via* MHC complexes on the surfaces of antigen-presenting cells. This is achieved by the enormous clonal diversity of TCRs generated by combining different CDRs within α and β TCR chains and by the pairing of differently combined α and β TCR chains (46). Carreno et al. showed that a dendritic cell vaccine increased naturally occurring neoantigen-specific immunity and promoted a diverse neoantigen-specific TCR repertoire in terms of both TCR-β usage and clonal composition, thus broadening the antigenic breadth and clonal diversity of antitumor immunity (47). To elucidate the molecular basis of the 5~10% failure rate of the hepatitis B vaccine, Yang et al. (48) conducted high-throughput sequencing and bioinformatics analysis of TRB CDR3 repertoires and found that the diversity of TRB CDR3 was significantly increased in responders compared to non-responders, which suggested that individuals with increased TCR diversity had better vaccine responses. Our results showed an increased TCR diversity after vaccination, suggesting that the SARS-CoV-2 inactivated vaccine elicits adaptive T cell immunity that can facilitate the recognition of multiple antigens.

Our study showed that the number of CD8^+^ MAIT cells was significantly increased in inactivated SARS-CoV-2 vaccine recipients. Furthermore, TCRs also underwent significant clone amplification. MAIT cells are unconventional innate-like T cells. They recognize antigens derived from the riboflavin biosynthetic pathway produced by a wide range of microbes and presented by the MHC class-I related (MR1) protein (49–51). Following activation, MAIT cells rapidly produce cytokines that include IFN-γ, TNF, IL-17, and IL-22 and mediate the cytolysis of infected cells, leading to the control of various infections. Innate cytokines, such as IL-12 and IL-18, can activate some MAIT cellular functions in an MR1-independent fashion, and enhance MAIT cell TCR-dependent activation. MR1-independent responses are likely important in MAIT cell responses to viral infections and in diseases driven by cytokine storms provoked by bacterial exotoxins. Human MAIT cells predominantly express the CD8α coreceptor (CD8^+^), with a smaller subset lacking both CD4 and CD8 (double-negative, DN). CD8^+^ MAIT cells have higher levels of IL-12 and IL-18 receptors. CD8^+^ MAIT cells display a higher diversity of T cell receptor repertoires than DN MAIT cells. Furthermore, CD8^+^ MAIT cells had significantly higher *GZMB*, *PRF1*, and *granulysin* levels than DN MAIT cells. These data indicate that peripheral blood CD8^+^ MAIT cells display higher baseline expression of coactivating receptors and cytotoxic effector molecules than DN MAIT cells (52). Provine et al. (53) showed that ChAdOx1 (chimpanzee adenovirus Ox1) immunization activated MAIT cells robustly. Activation of MAIT cells correlated with vaccine-induced T cell responses in human volunteers. MAIT cell-deficient mice displayed impaired CD8^+^ T cell responses to multiple vaccine-encoded antigens, suggesting that MAIT cells contribute to the immunogenicity of adenovirus-vectored vaccines. Boulouis et al. (54) found that pre-and postvaccination levels of MAIT cells correlated positively with the magnitude of SARS-CoV-2 spike protein-specific CD4^+^T cell and antibody responses in healthy vaccinees and that the MAIT cell compartment is involved in the early stages of priming of adaptive immune responses, which may be important for vaccine-induced immunity. Khaitan et al. (55) showed that HIV-infected children between the ages of 3 to 18 years have significantly decreased CD8^+^ MAIT cell populations compared to uninfected healthy children. CD8^+^ MAIT levels gradually increased with antiretroviral therapy, with greater recovery with the initiation of treatment at a younger age. Diminished CD8^+^ MAIT cell frequencies are associated with low CD4:CD8 ratios and elevated sCD14, suggesting a link with HIV disease progression. Moreover, CD8^+^ MAIT cell levels correlate tightly with other antibacterial and mucosa-protective immune subsets, namely, neutrophils, innate-like T cells, and Th17 and Th22 cells. These findings suggested that decreased MAIT cell populations in HIV-infected children are part of a concerted disruption of the innate and adaptive immune compartments specialized in sensing and responding to pathogenic or commensal bacteria.

Our study also showed that the differentiation as well as the number of CD8^+^ MAIT cells was promoted after inactivated SARS-CoV-2 vaccination. As shown in **Figure 4**, during the vaccination timeline, CD8^+^ MAIT cells advanced toward full differentiation. After the third injection, a large number of CD8^+^ MAIT cells were located at the endpoint of differentiation, indicating that three doses of inactivated SARS-CoV-2 vaccine significantly enhanced CD8^+^ MAIT cell differentiation, with the most enhancement after the third inoculation. Walker et al. (56) indicated that CD8^+^ MAIT cells are important tissue-homing cell populations, characterized by high expression of CD161 (++) and type-17 differentiation. By transcriptional and functional analyses, a pool of polyclonal, pre-committed type-17 CD161(++)CD8αβ(+) T cells circulate in cord blood, from which a prominent MAIT cell (TCR+) population emerges after postnatal antigen exposures and readily transitions to a CD8αα status in peripheral blood or at tissue sites. The potent cytokine secretion and homing pattern of expanded CD8^+^ MAIT-cell populations studied here suggest a central role in host defense and tissue inflammation in health and disease. To examine circulating MAIT cell levels and function in a healthy population, Lee et al. (57) enrolled 133 healthy subjects and measured MAIT cells and their subsets by flow cytometry. Circulating MAIT cell levels varied widely (0.19% to 21.7%) and were significantly lower in older individuals (age 61-92 years) than in young subjects (age 21-40 years). Although circulating MAIT cell levels were similar between male and female subjects, linear regression revealed that levels declined annually by 3.2% among men and 1.8% among women. Notably, the proportion of CD4^+^ MAIT cells increased, whereas that of CD8^+^ MAIT cells decreased with age. These studies showed that the proliferation and differentiation of CD8^+^ MAIT cells may be important markers of immune response. In our study, SARS-CoV-2 vaccination induced both CD8^+^MAIT cell proliferation and differentiation, indicating enhanced early immunity and rapid response to the target virus.

Our study found that NK cellular expressions of DEGs *KLRD1*, *GZMB*, and *PRF1* increased significantly after SARS-CoV-2 inactivated vaccination. At the same time, the expressions of these three DEGs by CD8^+^ effector T cells and CD8^+^ MAIT cells were also increased (**Figure 5**). Adaptive NK cells are currently grouped into three major categories, including cytokine-induced, memory-like, and true antigen-specific NK cells (58, 59). Cytokine-induced memory NK cells respond to specific cytokine profiles and seem to retain “memory” of a previous activation (60), memory-like NK cells are potent effector cells *via* antibody-dependent cellular cytotoxicity (61), and true antigen-specific NK cells respond to cytomegalovirus and adenoviral vaccine vectors in a peptide-specific manner and may utilize the NKG2C-CD94 heterodimer to identify specific target cells (62, 63). Bongen et al. (64) found that a gene associated with NK cells, *KLRD1*, which encodes CD94, was expressed at lower levels in symptomatic influenza virus shedders at baseline in discovery and validation cohorts. *KLRD1* expression in circulating NK cells at baseline negatively correlated with influenza susceptibility and symptom severity. In addition, *KLRD1* expression was positively correlated with several cytotoxic granule-associated genes, which included *PRF1* and those encoding granzymes (*GZMA*, *GZMB*, and *GZMH*), suggesting that higher *KLRD1* expression may correlate with increased proportions of cytotoxic cells. These results imply that *KLRD1*-expressing NK cells may serve as a novel biomarker for influenza susceptibility and that their early response may reduce and potentially prevent symptoms entirely. Our study also showed that *KLRD1* expression in NK cells increased significantly after inactivated SARS-CoV-2 vaccination. Furthermore, *GZMB* and *PRF1* expressions in T cells were also promoted. Therefore, whether the postvaccination upregulation of *KLRD1* expression can confer protection by promoting the expressions of *GZMB* and *PRF1*, and whether *KLRD1* expression is related to COVID-19 susceptibility needs to be clarified.

Our T cell enrichment pathway analysis found that nuclear-transcribed mRNA catabolic process, nonsense-mediated decay (NMD), and translational initiation were significantly upregulated after inactivated SARS-CoV-2 vaccination (**Figure 6**). NMD, which degrades flawed cellular mRNA by translating codons, not only contributes to a cellular quality control system to prevent abnormal protein synthesis but dynamically adapts the transcriptome and proteome to varying physiological conditions. The upregulation of NMD is closely related to the stress response. NMD confers positive-sense single-stranded RNA virus-restricting capacities, suggesting that the cellular RNA decay process may act as a primitive mechanism of intracellular antiviral immunity (65). In addition to its quality control function, which usually involves mRNA degradation, NMD also controls the abundance of ~10% of the cellular transcriptome (66). NMD has the capacity to co-regulate the abundance of entire groups of genes. Furthermore, as a post-transcriptional mechanism, NMD can facilitate rapid cellular responses to various stimuli. These processes are utilized during both cellular development and stress but may be circumvented by infecting viruses. The postvaccination upregulation of this pathway demonstrated in our study suggests that mechanisms of the SARS-CoV-2 vaccine may be related to quality control, limitation of SARS-CoV-2 replication, and rapid response.

Our study showed significant upregulation of the translation initiation T cell enrichment pathway after inactivated SARS-CoV-2 vaccination. Translation can initiate at alternate, non-canonical start codons in response to stressful stimuli. Viral infections and antiviral responses alter sites of translation initiation, and in some cases, lead to the production of novel immune epitopes. Machkovech et al. (67) showed that the subset of host transcripts induced by the antiviral response is enriched for alternate initiation sites upon influenza infection or during the antiviral response. Their results systematically mapped the landscape of translation initiation during influenza virus infection, and shed light on the evolutionary forces shaping this landscape. Thus, whether translation initiation promotes the production of protective antigen epitopes after inactivated SARS-CoV-2 vaccination deserves further study.

Our study showed that B cells did not proliferate after inactivated SARS-CoV-2 vaccination, while B-cell and B-cell receptor signaling enriched pathways were significantly upregulated, which is consistent with the study by Yin et al. (68). However, Yin et al. indicated that a SARS-CoV-2 inactivated vaccine induced activation of regulatory CD4^+^ T cells and CD8^+^ cytotoxic T cells, which may contribute to vaccine-induced T cell memory. The present study, however, focused on the vaccination-enhanced CD8^+^ MAIT cells, which enhanced early and quick immune response.

Several limitations of this study should be noted. Only three subjects were recruited. Follow-up tracked only to day 90 after the third inoculation. A larger population of subjects and longer follow-ups are required in subsequent studies to further characterize the overall immunological landscape after vaccination. Related molecular mechanisms need to be explored. In addition, the effects of innate immunity on adaptive immunity in the context of vaccination remain to be elucidated.

## Conclusion

Inactivated SARS-CoV-2 vaccination promoted T cell proliferation, TCR clone amplification, and TCR diversity, conferring adaptive T cell immunity to recognize a variety of antigens. The proliferation and differentiation of CD8^+^ MAIT cells and the *KLRD1* gene expression were significantly enhanced. Furthermore, the two pathways of the nuclear-transcribed mRNA catabolic process, NMD, and translational initiation were upregulated, which enhanced early immunity and rapid anti-viral responses.

## Data availability statement

The raw sequence data reported in this paper have been deposited in the Genome Sequence Archive (Genomics, Proteomics & Bioinformatics 2021) in National Genomics Data Center (Nucleic Acids Res 2022), China National Center for Bioinformation / Beijing Institute of Genomics, Chinese Academy of Sciences (GSA-Human: HRA005241) that are publicly accessible at https://ngdc.cncb.ac.cn/gsa-human.

## Ethics statement

The studies involving human participants were reviewed and approved by the Research Ethics Committee of the Third Affiliated Hospital of Shenzhen University. The patients/participants provided their written informed consent to participate in this study.

## Author contributions

XD and MP were major contributors in designing the study and writing the manuscript. RJ collected and analysed the data. WL and XZ designed the study and revised the article. All authors contributed to the article and approved the submitted version.

## References

[1] Zhu N Zhang D Wang W Li X Yang B Song J. China novel coronavirus investigating and research team. A novel coronavirus from patients with pneumonia in china, 2019. N Engl J Med (2020) 382(8):727–33. doi: 10.1056/NEJMoa2001017 PMC7092803 31978945

[2] Zhou P Yang XL Wang XG Hu B Zhang L Zhang W. A pneumonia outbreak associated with a new coronavirus of probable bat origin. Nature (2020) 579(7798):270–3. doi: 10.1038/s41586-020-2012-7 PMC7095418 32015507

[3] Coronaviridae Study Group of the International Committee on Taxonomy of Viruses. The species Severe acute respiratory syndrome-related coronavirus: classifying 2019-nCoV and naming it SARS-CoV-2. Nat Microbiol (2020) 5(4):536–44. doi: 10.1038/s41564-020-0695-z PMC7095448 32123347

[4] Li Q Guan X Wu P Wang X Zhou L Tong Y. Early transmission dynamics in wuhan, china, of novel coronavirus-infected pneumonia. N Engl J Med (2020) 382(13):1199–207. doi: 10.1056/NEJMoa2001316 PMC7121484 31995857

[5] Chan JF Yuan S Kok KH To KK Chu H Yang J. A familial cluster of pneumonia associated with the 2019 novel coronavirus indicating person-to-person transmission: a study of a family cluster. Lancet (2020) 395(10223):514–23. doi: 10.1016/S0140-6736(20)30154-9 PMC7159286 31986261

[6] Mahase E. China coronavirus: WHO declares international emergency as death toll exceeds 200. BMJ (2020) 368:m408. doi: 10.1136/bmj.m408 32005727

[7] Guo YR Cao QD Hong ZS Tan YY Chen SD Jin HJ. The rigin, transmission and clinical therapies on coronavirus disease 2019 (COVID-19) outbreak – an update on the status. Mil Med Res (2020) 7(1):11. doi: 10.1186/s40779-020-00240-0 32169119 PMC7068984

[8] Pollard CA Morran MP Nestor-Kalinoski AL. The COVID-19 pandemic: a global health crisis. Physiol Genomics (2020) 52(11):549–57. doi: 10.1152/physiolgenomics.00089.2020 PMC7686876 32991251

[9] Jackson LA Anderson EJ Rouphael NG Roberts PC Makhene M Coler RN. mRNA-1273 Study Group. An mRNA Vaccine against SARS-CoV-2 – Preliminary Report. N Engl J Med (2020) 383(20):1920–31. doi: 10.1056/NEJMoa2022483 PMC7377258 32663912

[10] Mulligan MJ Lyke KE Kitchin N Absalon J Gurtman A Lockhart S. Phase I/II study of COVID-19 RNA vaccine BNT162b1 in adults. Nature. (2020) 586(7830):589–93. doi: 10.1038/s41586-020-2639-4 32785213

[11] Folegatti PM Ewer KJ Aley PK Angus B Becker S Belij-Rammerstorfer S. Safety and immunogenicity of the ChAdOx1 nCoV-19 vaccine against SARS-CoV-2: a preliminary report of a phase 1/2, single-blind, randomised controlled trial. Lancet. (2020) 396(10249):467–78. doi: 10.1016/S0140-6736(20)31604-4 PMC7445431 32702298

[12] Wei J Stoesser N Matthews PC Ayoubkhani D Studley R Bell I. Antibody responses to SARS-CoV-2 vaccines in 45,965 adults from the general population of the United Kingdom. Nat Microbiol (2021) 6(9):1140–9. doi: 10.1038/s41564-021-00947-3 PMC8294260 34290390

[13] Sauré D O’Ryan M Torres JP Zuniga M Santelices E Basso LJ. Dynamic IgG seropositivity after rollout of CoronaVac and BNT162b2 COVID-19 vaccines in Chile: a sentinel surveillance study. Lancet Infect Dis (2022) 22(1):56–63. doi: 10.1016/S1473-3099(21)00479-5 34509185 PMC8428469

[14] Kramer KJ Wilfong EM Voss K Barone SM Shiakolas AR Raju N. Single-cell profiling of the antigen-specific response to BNT162b2 SARS-coV-2 RNA vaccine. Nat Commun . (2022) 13(1):3466. doi: 10.1038/s41467-022-31142-5 35710908 PMC9201272

[15] Cao Q Wu S Xiao C Chen S Chi X Cui X. Integrated single-cell analysis revealed immune dynamics during Ad5-nCoV immunization. Cell Discovery (2021) 7(1):64. doi: 10.1038/s41421-021-00300-2 34373443 PMC8352953

[16] Zhang JY Wang XM Xing X Xu Z Zhang C Song JW. Single-cell landscape of immunological responses in patients with COVID-19. Nat Immunol (2020) 21(9):1107–18. doi: 10.1038/s41590-020-0762-x 32788748

[17] Chen S Zhou Y Chen Y Gu J. fastp: an ultra-fast all-in-one FASTQ preprocessor. Bioinformatics (2018) 34(17):i884–90. doi: 10.1093/bioinformatics/bty560 PMC6129281 30423086

[18] Martin M. Cutadapt removes adapter sequences from high-throughput sequencing reads. EMBnet J (2011) 17:10–2. doi: 10.14806/ej.17.1.200

[19] Dobin A Davis CA Schlesinger F Drenkow J Zaleski C Jha S. STAR: ultrafast universal RNA-seq aligner. Bioinformatics. (2013) 29(1):15–21. doi: 10.1093/bioinformatics/bts635 23104886 PMC3530905

[20] Liao Y Smyth GK Shi W. featureCounts: an efficient general purpose program for assigning sequence reads to genomic features. Bioinformatics. (2014) 30(7):923–30. doi: 10.1093/bioinformatics/btt656 24227677

[21] Satija R Farrell JA Gennert D Schier AF Regev A. Spatial reconstruction of single-cell gene expression data. Nat Biotechnol (2015) 33(5):495–502. doi: 10.1038/nbt.3192 25867923 PMC4430369

[22] Korsunsky I Millard N Fan J Slowikowski K Zhang F Wei K. Fast, sensitive and accurate integration of single-cell data with Harmony. Nat Methods (2019) 16(12):1289–96. doi: 10.1038/s41592-019-0619-0 PMC6884693 31740819

[23] Yu G Wang LG Han Y He QY. clusterProfiler: an R package for comparing biological themes among gene clusters. OMICS. (2012) 16(5):284–7. doi: 10.1089/omi.2011.0118 PMC3339379 22455463

[24] Hodgson SH Mansatta K Mallett G Harris V Emary KRW Pollard AJ. What defines an efficacious COVID-19 vaccine? A review of the challenges assessing the clinical efficacy of vaccines against SARS-CoV-2. Lancet Infect Dis (2021) 21(2):e26–35. doi: 10.1016/S1473-3099(20)30773-8 PMC7837315 33125914

[25] Dai L Gao GF. Viral targets for vaccines against COVID-19. Nat Rev Immunol (2021) 21(2):73–82. doi: 10.1038/s41577-020-00480-0 33340022 PMC7747004

[26] Chung JY Thone MN Kwon YJ. COVID-19 vaccines: The status and perspectives in delivery points of view. Adv Drug Delivery Rev (2021) 170:1–25. doi: 10.1016/j.addr.2020.12.011 PMC7759095 33359141

[27] Baden LR ElSahly HM Essink B Kotloff K Frey S Novak R. Efficacy and safety of the mRNA-1273 SARS-coV-2 vaccine. N Engl J Med (2021) 384(5):403–16. doi: 10.1056/NEJMoa2035389 PMC7787219 33378609

[28] Polack FP Thomas SJ Kitchin N Absalon J Gurtman A Lockhart S. Safety and efficacy of the BNT162b2 mRNA covid-19 vaccine. N Engl J Med (2020) 383(27):2603–15. doi: 10.1056/NEJMoa2034577 PMC7745181 33301246

[29] Dagan N Barda N Kepten E Miron O Perchik S Katz MA. BNT162b2 mRNA covid-19 vaccine in a nationwide mass vaccination setting. N Engl J Med (2021) 384(15):1412–23. doi: 10.1056/NEJMoa2101765 PMC7944975 33626250

[30] Haas EJ Angulo FJ McLaughlin JM Anis E Singer SR Khan F. Impact and effective- ness of mRNA BNT162b2 vaccine against SARS-CoV-2 infections and COVID-19 cases, hospitalisations, and deaths following a nation- wide vaccination campaign in Israel: an observational study using national surveillance data. Lancet. (2021) 397(10287):1819–29. doi: 10.1016/S0140-6736(21)00947-8 PMC8099315 33964222

[31] Tenforde MW Olson SM Self WH Talbot HK Lindsell CJ Steingrub JS. Effectiveness of pfizer-bioNTech and moderna vaccines against COVID-19 among hospitalized adults aged ≥65 years – united states, january-march 2021. MMWR Morb Mortal Wkly Rep (2021) 70(18):674–9. doi: 10.15585/mmwr.mm7018e1 PMC9368749 33956782

[32] Sadoff J LeGars M Shukarev G Heerwegh D Truyers C Groot AM. Interim results of a phase 1-2a trial of ad26.COV2.S covid-19 vaccine. N Engl J Med (2021) 384(19):1824–35. doi: 10.1056/NEJMoa2034201 PMC7821985 33440088

[33] Voysey M Clemens SAC Madhi SA Weckx LY Folegatti PM Aley PK. Safety and efficacy of the ChAdOx1 nCoV-19 vaccine (AZD1222) against SARS-CoV-2: an interim analysis of four randomised controlled trials in Brazil, South Africa, and the UK. Lancet. (2021) 397(10269):99–111. doi: 10.1016/S0140-6736(20)32661-1 33306989 PMC7723445

[34] Keehner J Horton LE Pfeffer MA Longhurst CA Schooley RT Currier JS. SARS-coV-2 infection after vaccination in health care workers in california. N Engl J Med (2021) 384(18):1774–5. doi: 10.1056/NEJMc2101927 PMC8008750 33755376

[35] Juthani PV Gupta A Borges KA Price CC Lee AI Won CH. Hospitalisation among vaccine breakthrough COVID-19 infections. Lancet Infect Dis (2021) 21(11):1485–6. doi: 10.1016/S1473-3099(21)00558-2 PMC8423430 34506735

[36] Bosch W Cowart JB Bhakta S Carter RE Wadei HM Shah SZ. COVID-19 vaccine-breakthrough infections requiring hospitalization in mayo clinic florida through august 2021. Clin Infect Dis (2022), 75(1):e892–e894. doi: 10.1093/cid/ciab932 PMC8689905 34726700

[37] Brosh-Nissimov T Orenbuch-Harroch E Chowers M Elbaz M Nesher L Stein M. BNT162b2 vaccine breakthrough: clinical characteristics of 152 fully vacci- nated hospitalized COVID-19 patients in Israel. Clin Microbiol Infect (2021) 27(11):1652–7. doi: 10.1016/j.cmi.2021.06.036 PMC8261136 34245907

[38] Duarte LF Gálvez NMS Iturriaga C Melo-González F Soto JA Schultz BM. Immune profile and clinical outcome of breakthrough cases after vaccination with an inactivated SARS-coV-2 vaccine. Front Immunol (2021) 12:742914. doi: 10.3389/fimmu.2021.742914 34659237 PMC8511644

[39] Fiolet T Kherabi Y MacDonald CJ Ghosn J Peiffer-Smadja N. Comparing COVID-19 vaccines for their characteristics, efficacy and effectiveness against SARS-CoV-2 and variants of concern: a narrative review. Clin Microbiol Infect (2022) 28(2):202–21. doi: 10.1016/j.cmi.2021.10.005 PMC8548286 34715347

[40] Jiang R Dou X Li M Wang E Hu J Xiong D. Dynamic observation of SARS-CoV-2 IgM, IgG, and neutralizing antibodies in the development of population immunity through COVID-19 vaccination. J Clin Lab Anal (2022) 36(4):e24325. doi: 10.1002/jcla.24325 35235705 PMC8993648

[41] Hao Y Hao S Andersen-Nissen E Mauck WM Zheng S Butler A. Integrated analysis of multimodal single-cell data. Cell. (2021) 184(13):3573–3587.e29. doi: 10.1016/j.cell.2021.04.048 34062119 PMC8238499

[42] Zhang H Hu Y Jiang Z Shi N Lin H Liu Y. Single-cell sequencing and immune function assays of peripheral blood samples demonstrate positive responses of an inactivated SARS-CoV-2 vaccine. Lancet (2021). doi: 10.2139/ssrn3774153

[43] Horns F Dekker CL Quake SR. Memory B cell activation, broad anti-influenza antibodies, and bystander activation revealed by single-cell transcriptomics. Cell Rep (2020) 30(3):905–913.e6. doi: 10.1016/j.celrep.2019.12.063 31968262 PMC7891556

[44] Kong L Moorlag SJ Lefkovith A Li B Matzaraki V Emst L. Single-cell transcriptomic profiles reveal changes associated with BCG-induced trained immunity and protective effects in circulating monocytes. Cell Rep (2021) 37(7):110028. doi: 10.1016/j.celrep.2021.110028 34788625 PMC8728743

[45] Mateus J Dan JM Zhang Z Moderbacher CR Lammers M Goodwin B. Low-dose mRNA-1273 COVID-19 vaccine generates durable memory enhan- ced by cross-reactive T cells. Science. (2021) 374(6566):eabj9853. doi: 10.1126/science.abj9853 34519540 PMC8542617

[46] Wing JB Sakaguchi S. TCR diversity and Treg cells, sometimes more is more. Eur J Immunol (2011) 41(11):3097–100. doi: 10.1002/eji.201142115 22015985

[47] Carreno BM Magrini V Becker-Hapak M Kaabinejadian S Hundal J Petti AA. Cancer immunotherapy. A dendritic cell vaccine increases the breadth and diversity of melanoma neoantigen-specific T cells. Sci . (2015) 348(6236):803–8. doi: 10.1126/science.aaa3828 PMC4549796 25837513

[48] Yang J Li Y Ye J Wang J Lu H Yao X. Characterization of the TCR β Chain repertoire in peripheral blood from hepatitis B vaccine responders and non-responders. J Inflammation Res (2022) 15:939–51. doi: 10.2147/JIR.S347702 PMC8856041 35210805

[49] Corbett AJ Eckle S Birkinshaw RW Liu L Patel O Mahony J. T-cell activation by transitory neo-antigens derived from distinct microbial pathways. Nature. (2014) 509(7500):361–5. doi: 10.1038/nature13160 24695216

[50] Kjer-Nielsen L Patel O Corbett AJ Nours JL Meehan B Liu L. MR1 presents microbial vitamin B metabolites to MAIT cells. Nature. (2012) 491(7426):717–23. doi: 10.1038/nature11605 23051753

[51] Bourhis LL Martin E Péguillet I Guihot A Froux N Coré M. Antimicrobial activity of mucosal-associated invariant T cells. NatImmunol. (2010) 11(8):701–8. doi: 10.1038/ni.1890 20581831

[52] Dias J Boulouis C Gorin JB Biggelaar R Lal KG Gibbs A. The CD4 ^–^ CD8 ^–^ MAIT cell subpopulation is a functionally distinct subset developmentally related to the main CD8 ^+^ MAIT cell pool. Proc Natl Acad Sci U.S.A. (2018) 115(49):E11513–22. doi: 10.1073/pnas.1812273115 PMC6298106 30442667

[53] Provine NM Amini A Garner LC Spencer AJ Dold C Hutchings C. MAIT cell activation augments adenovirus vector vaccine immunogenicity. Science. (2021) 371(6528):521–6. doi: 10.1126/science.aax8819 PMC7610941 33510029

[54] Boulouis C Kammann T Cuapio A Parrot T Gao Y Mouchtaridi E. MAIT cell compartment characteristics are associated with the immune response magnitude to the BNT162b2 mRNA anti-SARS-CoV-2 vaccine. Mol Med (2022) 28(1):54. doi: 10.1186/s10020-022-00484-7 35562666 PMC9100314

[55] Khaitan A Kilberg M Kravietz A Ilmet T Tastan C Mwamzuka M. HIV-infected children have lower frequencies of CD8+ Mucosal-associated invariant T (MAIT) cells that correlate with innate, th17 and th22 cell subsets. PloS One (2016) 11(8):e0161786. doi: 10.1371/journal.pone.0161786 27560150 PMC4999196

[56] Walker LJ Kang YH Smith MO Tharmalingham H Ramamurthy N Fleming VM. Human MAIT and CD8αα cells develop from a pool of type-17 precommitted CD8+ T cells. Blood. (2012) 119(2):422–33. doi: 10.1182/blood-2011-05-353789 PMC3257008 22086415

[57] Lee OJ Cho YN Kee SJ Kim MJ Jin HM Lee SJ. Circulating mucosal-associated invariant T cell levels and their cytokine levels in healthy adults. Exp Gerontol. (2014) 49:47–54. doi: 10.1016/j.exger.2013.11.003 24269212

[58] Paust S Blish CA Reeves RK. Redefining memory: building the case for adaptive NK cells. J Virol (2017) 91(20):e00169–17. doi: 10.1128/JVI.00169-17 PMC5625515 28794018

[59] Ram DR Lucar O Hueber B Reeves RK. Simian immunodeficiency virus infection modu- lates CD94 ^+^ (*KLRD1* ^+^) NK cells in rhesus macaques. J Virol (2019) 93(16):e00731–19. doi: 10.1128/JVI.00731-19 PMC6675877 31167916

[60] Ghofrani J Lucar O Dugan H Reeves RK Jost S. Semaphorin 7A modulates cytokine- induced memory-like responses by human natural killer cells. Immunol. (2019) 49(8):1153–1166. doi: 10.1002/eji.201847931 PMC6679804 31016720

[61] Zhang T Scott JM Hwang I Kim S. Cutting edge: antibody-dependent memory-like NK cells distinguished by FcRγ deficiency. J Immunol (2013) 190(4):1402–6. doi: 10.4049/jimmunol.1203034 PMC3623944 23345329

[62] Reeves RK Li H Jost S Blass E Li H Schafer JL. Antigen-specific NK cell memory in rhesus macaques. Nat Immunol (2015) 16(9):927–32. doi: 10.1038/ni.3227 PMC4545390 26193080

[63] Hammer Q Rückert T Borst EM Dunst J Haubner A Durek P. Peptide-specific recognition of human cytomegalovirus strains controls adaptive natural killer cells. Nat Immunol (2018) 19(5):453–63. doi: 10.1038/s41590-018-0082-6 29632329

[64] Bongen E Vallania F Utz PJ Khatri P. *KLRD1*-expressing natural killer cells predict influenza susceptibility. Genome Med (2018) 10(1):45. doi: 10.1186/s13073-018-0554-1 29898768 PMC6001128

[65] Tran GVQ Kleinehr J Preugschas HF Anhlan D Mohamed FF Ehrhardt C. Nonsense-mediated mRNA decay does not restrict influenza A virus propagation. Cell Microbiol (2021) 23(6):e13323. doi: 10.1111/cmi.13323 33655690

[66] Kurosaki T Popp MW Maquat LE. Quality and quantity control of gene expression by nonsense-mediated mRNA decay. Nat Rev Mol Cell Biol (2019) 20(7):406–20. doi: 10.1038/s41580-019-0126-2 PMC6855384 30992545

[67] Machkovech HM Bloom JD Subramaniam AR. Comprehensive profiling of translation initiation in influenza virus infected cells. PloS Pathog (2019) 15(1):e1007518. doi: 10.1371/journal.ppat.1007518 30673779 PMC6361465

[68] Yin J Zhao Y Huang F Yang Y Huang Y Zhuang Z. Immune response and homeostasis mechanism following administration of BBIBP-CorV SARS-CoV-2 inactivated vaccine. Innovation (Camb) (2023) 4(1):100359. doi: 10.1016/j.xinn.2022.100359 36506806 PMC9719934

